# Correlation Between Immunohistochemical Biomarkers Expression and Prognosis of Ovarian Carcinomas in Tunisian Patients

**DOI:** 10.4021/wjon2010.06.213w

**Published:** 2010-05-19

**Authors:** Lobna Ayadi, Salma Chaabouni, Abdelmajid Khabir, Habib Amouri, Saloua Makni, Mohamed Guermazi, Mounir Frikha, Tahya Sellami Boudawara

**Affiliations:** a Department of Pathology, Habib Bourguiba University Hospital, Sfax, Tunisia; b Department of Gynecology, Hedi Chaker University Hospital, Sfax, Tunisia; c Department of Oncology, Habib Bourguiba University Hospital, Sfax, Tunisia

**Keywords:** Ovarian carcinoma, p53, Bcl-2, Estrogen receptor, Progesterone receptor

## Abstract

**Background:**

Ovarian cancer is the leading cause of death from gynaecological malignancies. Newer biological prognostic factors and predictors of response to therapy are needed. Our study was designed to evaluate the expression of p53, Bcl-2, Estrogen receptor (ER) and Progesterone receptor (PR) in ovarian carcinoma and to compare it with other prognostic parameters such as age, FIGO stage, size of residual tumor, histological type and grade.

**Methods:**

This is a retrospective study conducted in the department of pathology at Sfax University Hospital. Confirmed 57 cases of ovarian carcinoma were reviewed in the period between January 1995 and December 2006. We used immunohistochemistry to evaluate the expression of p53, Bcl-2, ER and PR receptors and Chi-Square and Student test to correlate immunohistochemical findings with some prognostic parameters of ovarian carcinoma.

**Results:**

The percentage of expression of p53, Bcl-2, ER and PR was 73,7; 47,4; 35,1 and 33,3 % respectively. p53 overexpression correlated with an advanced FIGO stage (p = 0,026) and presence of ascitis (p < 10^-4^). The expression of PR was associated with an early stage (FIGO I and II), a non serous histologic type and a low tumour grade (p = 0,045; 0,010 and 0,036 respectively). No correlation was found between Bcl-2 and ER and prognostic parameters. Survival analysis revealed that Bcl-2 status, FIGO stage, presence of ascites, peritoneal cytology, and residual disease were significant predictive factors of survival.

**Conclusion:**

p53 expression correlates with a worse prognosis in epithelial ovarian cancer, whereas Bcl-2 expression is related to a better outcome. For hormonal status, expression of PR is found to be an independent indicator of favourable prognosis. These results should be supported by more and larger studies.

## Introduction

Epithelial ovarian carcinoma is worldwide the sixth most common female cancer [1]. This malignancy carries the highest mortality among all gynaecological cancers [2, 3]. According to data from the Cancer Registry of Tunisia, ovarian carcinoma is the second gynaecological cancer with an incidence of 2.9/100000 inhabitants [4]. Identification of new biological prognostic markers would be of great importance to select patients with a possibly favourable or poor clinical outcome and might help to improve treatment planning [5].

Regulators of apoptosis, especially p53 and Bcl-2, and steroid hormone receptors, estrogen and progesterone, have been studied as potential prognostic factors of epithelial ovarian cancer.

P53 is a tumor suppressor gene located on the short arm of chromosome 17. Mutation of p53 is believed to result in uncontrolled cell proliferation [6]. Mutant p53 protein may be identified by immunohistochemical methods, related to the longer time required for the destruction of the mutant protein compared to the wild type [7].

The Bcl-2 gene, a member of the Bcl-2 family inhibits apoptosis and has been shown to exert antiapoptotic activity in ovarian carcinoma cells responding to chemotherapy [8].

Steroid hormones are thought to play an important role in the process of carcinogenesis in ovarian carcinoma. Estrogen may contribute to initiation and/or promotion of ovarian carcinogenesis. It is thus logical to speculate that the overexpression of ER should be associated with a poor prognosis [9]. On the other hand, progesterone may offer protection against ovarian carcinoma development.

The purposes of the present study were to study the immunohistochemical profile of p53, Bcl-2 and steroid hormone receptors in a series of 57 ovarian carcinomas, and to investigate their association with clinicopathological prognostic indicators.

## Materials and Methods

### Patients and specimens

In this study, we conducted a retrospective analysis of 69 epithelial ovarian cancers collected in the Sfax University Hospital between January 1995 and December 2006. Our study concerned a sample size of 57 because these were the only cases for which we had complete information about the patient and the tumor. Also, these were the only cases whose paraffin blocks had enough tissue to allow extra sections for our study and eventually for future examination. Borderline tumors were not included in this study.

In fact, epithelial ovarian cancer samples were studied after informed consent and IRB approval from the 57 patients. The patients’ age ranged from 29 to 77 years (mean age = 54 years). A total of 31 (54.4%) patients were 55 years old or less and 26 (45.6%) were older than 55 years. The FIGO 1988 classification was used in the database. Twenty (35.1%) patients were in early FIGO stage (I/II) and 37 (64.9 %) in later stage (III/IV). The histological type was determined on tissue sections according to World Health Organization criteria. The microscopic grading of Shimizu and Silverberg [10] was used: 15 cases were grade I, 19 cases grade II and 23 cases grade III.

The standard surgical procedure was: total abdominal hysterectomy, bilateral salpingo-oophorectomy, omentectomy, appendicectomy, multiple peritoneal biopsies and peritoneal washings with cytology. All patients received initial cytoreductive surgery which was complete or optimal in 31 cases (54.4 %) and suboptimal in 26 (45.6 %).

Patients were treated according to a standardized adjuvant chemotherapy protocol (Cisplatin: 50 mg/m^2^ and Cyclophosphamide: 500 mg/m^2^ in combination in six courses given every three weeks. To assess response, reports from first surgery and second look laparotomy were reviewed and all tumor measurements were compared. Complete pathologic response was defined as the disappearance of all tumor at second-look laparotomy, with all biopsy specimens and peritoneal washings negative for tumor cells. Microscopic residual disease was defined as the disappearance of all macroscopic tumor lesions but the presence of tumor cells in one or more biopsy specimens or peritoneal washings. Partial response was defined as a 50% or more decrease in size of all bidimensionally measured tumor lesions. Stable disease was either a decrease in size of less than 50% or an increase in size of less than 25% of one or more measured tumor lesions. Progressive disease was defined as either a 25% or more increase in the size of one or more clinically measured lesions or the appearance of new disease manifestations or a 25% increase in size of one or more tumor lesions at second-look laparotomy. All patients were followed up until death or August 2008. Follow-up information was collected from the medical records, and no patients were lost of follow-up. The median follow-up time for patients still alive was 31 months (range: 0-151 months). A detailed description of patient characteristics is given in Table 1.

**Table 1:** Clinicopathological data (n = 57)

	*N*	%
Age		
≤ 55 years	31	54.4
> 55 years	26	45.6
FIGO Stage		
I/II	20	35.1
III/IV	37	64.9
Ascites		
<100 ml	23	65.2
>100 ml	34	79.2
Cytology		
Negative	28	71.4
Positive	29	75.9
Residual disease		
None/Optimal	31	54.4
Suboptimal	26	45.6
Histologic type		
Serous	24	42.1
Non serous	33	57.9
Tumor grade		
I/II	34	59.6
III	23	40.4
p53		
Negative	15	26.3
Positive	42	73.7
Bcl-2		
Negative	30	52.6
Positive	27	47.4
ER		
Negative	37	64.9
Positive	20	35.1
PR		
Negative	38	66.7
Positive	19	33.3
Surgery		
Optimal	31	54.4
Suboptimal	26	45.6
Systemic chemotherapy		
Adjuvant	45	96.49
Neoadjuvant	2	3.51
Response		
CPR and micro only	28	49.1
Partial	2	3.5
Stable and progression	15	26.3
Not assessable	12	21.0

ER: Estrogen receptor; PR: Progesterone receptor; CPR: complete pathologic response; micro only: microscopic residual disease only.

### Pathological diagnosis

All surgical tissue specimens were fixed in 10% formaldehyde, embedded in paraffin, sectioned and stained with hematoxylin/eosin. According to the WHO classification [11], histology revealed serous carcinomas in 24 (42.1%), endometrioid carcinomas in 23 (40.3%), mucinous carcinomas in 3 (5.3%) and other histological types in 7 cases (12.3%) (Table 2).

**Table 2:** Histologic Subtypes

Histologic subtypes	Number	%
Serous carcinoma	24	42.1
Endometrioid carcinoma	23	40.3
Malignant mixed epithelial tumour	4	7
Mucinous carcinoma	3	5.3
Undifferenciated carcinoma	2	3.5
Transitional cell carcinoma	1	1.8

### Immunohistochemical staining

Immunostaining for p53, Bcl-2, ER and PR was performed for all specimens. Four micrometer sections attached on silanized slides were de-waxed in xylene, rehydrated in graded ethanol and covered with 10 mM citrate buffer (pH 6). They were then incubated for 30 min with primary monoclonal antibodies against p53 (DAKO, clone DO-7; 1:50), Bcl-2 (DAKO, clone 124; 1:100), ER (DAKO, clone 1D5; 1:25) and PR (DAKO, clone PgR636; 1:50), followed by incubation with biotin-labeled secondary antibodies. The streptavidin-peroxidase complex was visualized using di-aminobenzidine as a chromogenic substrate.

All slides were reviewed by the senior author with the supervision of one of the co-authors (AK) without knowledge of the clinical outcome. As positive controls, previously established positive cases of human tumors for p53, Bcl-2, ER and PR were used. For p53, ER and PR, only nuclear staining of the tumor cells was considered a positive expression. Bcl-2 immunostaining was typically cytoplasmic.

A semi-quantitative immunoreactive score was used to record results. The score is obtained by the product of intensity of immunostaining (none = 0; weak = 1; moderate = 2; strong = 3) and the percentage of positive tumor cells (none = 0; 1-25% = 1; 26-50% = 2; 51-75% = 3; >75% = 4).

### Statistical analysis

Statistical analysis was used to evaluate correlations between expression of p53, Bcl-2, ER and PR and clinicopathological parameters. Survival analysis was calculated using the method of Kaplan-Meier. It was done using the SPSS Inc software (version 11). Relationship between qualitative parameters was determined using the Chi-Square and Student tests. Statistical significance was defined as p < 0.05.

## Results

### Relationship between p53, Bcl-2 and ER, PR expression

**Figure 1:**
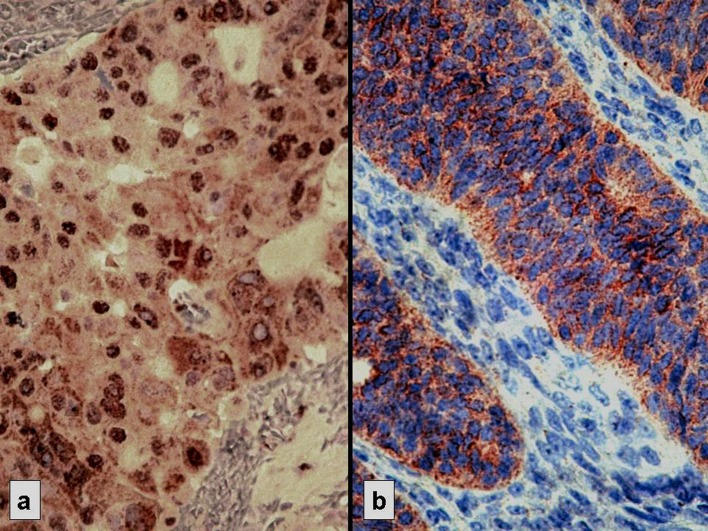
Strong immunostaining: nuclear for p53 (a) and cytoplasmic for Bcl-2 (b)

**Figure 2:**
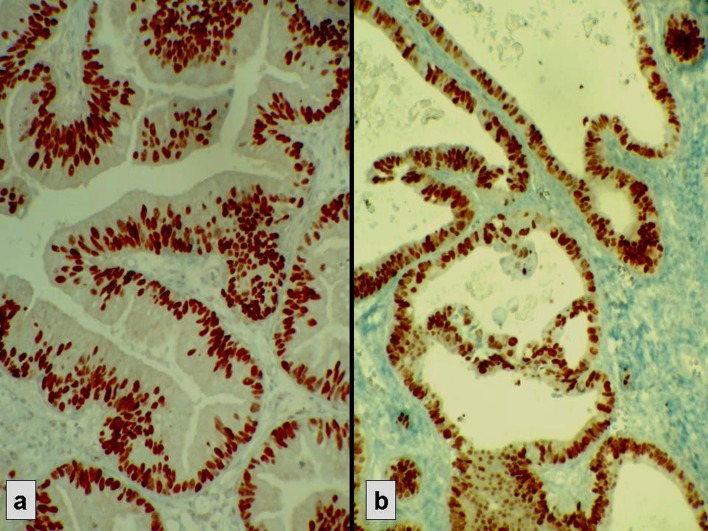
Strong nuclear immunostaining with hormonal receptors. (a) ER+, (b) PR+.

The expression rate of p53, Bcl-2, ER and PR was respectively 73.7; 47.4; 35.1; and 33.3% (Fig. 1 and 2). In most cases, ER and PR were both detected or both absent. This association was statistically significant (p = 0,000) (Table 3). No significant association was found between p53 and Bcl-2 status (Table 4).

**Table 3:** Correlation Between ER and PR Expression (p = 0.000)

	ER		Total
	Negative	Positive	
PR			
Negative	33 (86.8%)	5 (13.2%)	38
Positive	4 (21.1%)	15 (78.9%)	19
Total	37	20	57

ER: Estrogen receptor; PR: Progesterone receptor.

**Table 4:** Correlation Between p53 and Bcl-2 Expression (p = 0.949)

	p53		Total
	Negative	Positive	
Bcl-2			
Negative	8 (26.7%)	22 (73.3%)	30
Positive	7 (25.9%)	20 (74.1%)	27
Total	15	42	57

### Relationship between p53, Bcl-2 and ER and PR status and clinicopathological parameters

The associations between protein expression and the clinicopathologic parameters are shown in Tables 5 and 6.

**Table 5:** Correlation Between P53 and Bcl-2 Expression With Clinico-pathological Data

	N	P53 Expression				Bcl-2 Expression			
		%	p^1^	Score ± SD	p^2^	%	p^1^	Score ± SD	p^2^
Age (years)			0.611		0.372		0.153		0.230
≤ 55	31	71		3.23 ± 3.48		38.7		0. 84 ± 1.65	
>55	26	76.9		4.08 ± 3.64		57.7		1.42 ± 1.98	
FIGO Stage			0.085		0.026		0.169		0.893
I-II	20	60		2.20 ± 3.25		35		1.15 ± 2.27	
III-IV	37	81.5		4.38 ± 3.50		54.1		1.08 ± 1.55	
Residual disease			0.611		0.135		0.716		0.970
None/Optimal	31	71		2.97 ± 3.33		45.2		1.1 ± 1.98	
Sub-optimal	26	76.9		4.38 ± 3.71		50		1.12 ± 1.63	
Histologic type			0.847		0.320		0.462		0.271
Serous	24	75		4.17 ± 3.66		41.7		0.79 ± 1.35	
No Serous	33	72.7		3.21 ± 3.46		51.5		1.33 ± 2.08	
Histologic Grade			0.061		0.063		0.629		0.636
I-II	34	64.7		1.65 ± 0.48		50		1.50 ± 0.50	
III	23	87		1.87 ± 0.34		43.5		1.43 ± 0.50	
Ascites									
<100 ml	23	65.2	0.230	3.35 ± 3.74	0.646	39.1	0.306	0.83 ± 1.58	0.345
>100 ml	34	79.2		3.79 ± 3.45		52.9		1.29 ± 1.96	
Cytology					0.452		0.503		0.892
negative	28	71.4	0.700	3.25 ± 3.58		42.9		1.07 ± 1.96	
positive	29	75.9		3.97 ± 3.54		51.7		1.14 ± 1.70	

p^1^: Chi-Square test; p^2^: Student test; %: Positivity percentage; SD: standard deviation.

**Table 6:** Correlation Between ER and PR Status With Clinico-pathological Data

	N	ER Expression				PR Expression			
		%	p^1^	Score ± SD	p^2^	%	p^1^	Score ± SD	p^2^
Age (years)			0.296		0.614		0.707		0.537
≤ 55	31	29		1.06 ± 2.30		35.5		1.87 ± 3.05	
>55	26	42.3		1.38 ± 2.45		30.8		1.38 ± 2.80	
FIGO Stage			0.568		0.105		0.170		0.045
I-II	20	40		1.90 ± 3.38		45		2.70 ± 3.68	
III-IV	37	32.4		0.84 ± 1.48		27		1.08 ± 2.27	
Residual disease			0.945		0.289		0.039		0.013
None/Optimal	31	35.5		1.52 ± 2.89		45.2		2.52 ± 3.45	
Sub-optimal	26	34.6		0.85 ± 1.46		19.2		0.62 ± 1.67	
Histologic type			0.813		0.570		0.088		0.010
Serous	24	33.3		1.00 ± 1.64		20.8		0.50 ± 1.06	
No Serous	33	36.4		1.36 ± 2.78		42.4		2.48 ± 3.52	
Histologic Grade			0.021		0.021		0.036		0.036
I-II	34	47.1		1.47 ± 0.50		44.1		1.44 ± 0.50	
III	23	17.4		1.17 ± 0.38		17.4		1.17 ± 0.38	
Ascites			0.599		0.986		0.84		0.933
<100 ml	23	39.1		1.22 ± 1.70		34.8		1.61 ± 2.72	
>100 ml	34	32.4		1.21 ± 2.73		32.4		1.68 ± 3.09	
Cytology			0.70		0.90		0.134		0.182
Negative	28	71.4		1.75 ± 3.02		42.9		2.18 ± 3.19	
Positive	29	75.9		0.69 ± 1.31		24.1		1.14 ± 2.58	

p^1^: Chi-Square test; p^2^: Student test; %: positivity percentage; ER: Estrogen receptor; PR: Progesterone receptor; SD: standard deviation.

A statistically significant positive correlation was observed between p53 expression and advanced disease (FIGO stage III/IV) (p = 0.026) and the presence of ascites at the time of staging laparotomy (p < 10^-4^). There was no significant correlation between p53 expression and age, size of residual disease, histologic type and grade. PR expression was associated with an early FIGO stage (p = 0.045), a complete/optimal primary surgery (p = 0.013), a non serous histologic type (p = 0.010) and a low tumor grade (p = 0.036). No association was found between Bcl-2 and ER expression and the other clinicopathologic parameters studied.

At assessment on August 2008, 18 patients (31.5%) were alive without evidence of disease, 9 (15.7%) were alive with disease, 30 (52.6%) had died of ovarian cancer.

**Figure 3:**
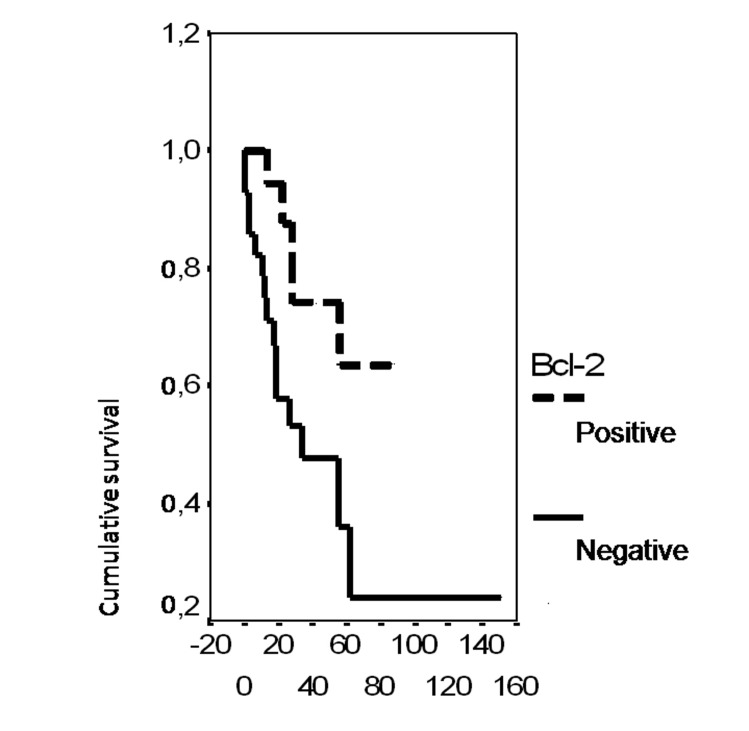
Cumulative survival probability in relation to Bcl-2 status.

**Figure 4:**
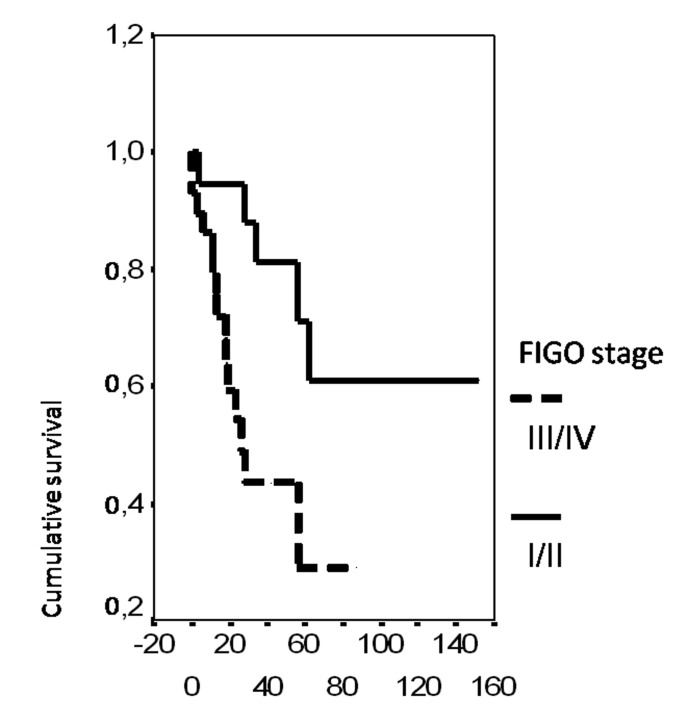
Cumulative survival probability in relation to FIGO stage.

**Figure 5:**
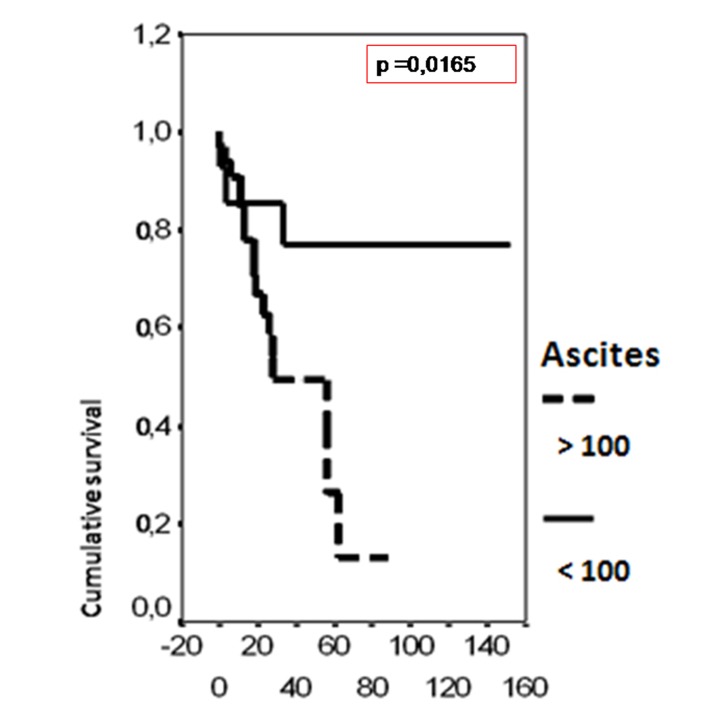
Cumulative survival probability in relation to presence of ascites.

**Figure 6:**
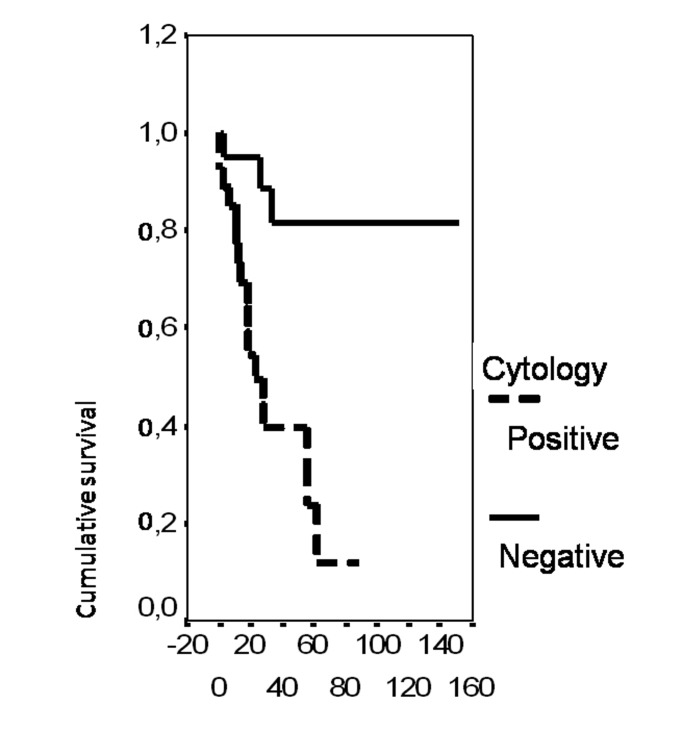
Cumulative survival probability in relation to peritoneal cytology.

**Figure 7:**
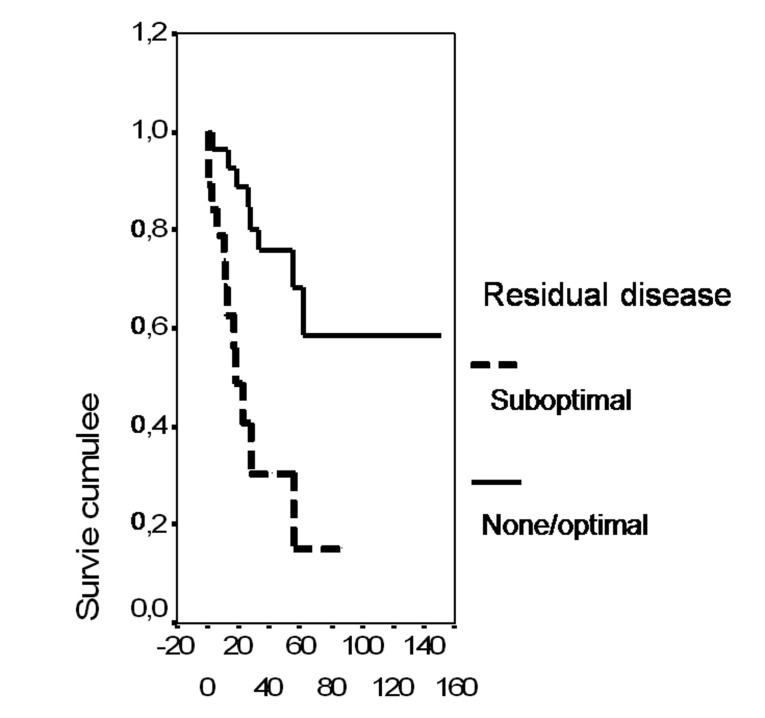
Cumulative survival probability in relation to residual disease.

In univariate analysis, the overall survival rate was significantly associated with the bcl-2 status (p = 0.02), the FIGO stage (p = 0.0135), the presence of ascites (p = 0.0165), peritoneal cytology status (p = 0.0004) and the size of residual disease (p = 0.0006). The results of analysis of the importance of clinicopathological parameters with regards to overall survival are presented in Figures 3-7.

## Discussion

The importance of p53 accumulation as a marker of adverse outcome in ovarian carcinoma has been demonstrated in several studies. Expression of p53 is associated with other unfavourable prognostic factors such as advanced FIGO stage, suboptimal cytoreduction, serous histologic subtype and increasing tumor grade. Nevertheless, its independent prognostic value remains controversial [6, 12]. Some investigators have demonstrated that p53 mutation or overexpression is a significant prognostic factor [13-15]. Other studies have been unable to confirm such results [16-18]. In our series, the p53 status was associated with FIGO stage.

In contrast to p53, Bcl-2 staining is most frequently associated with favourable pathologic parameters: endometrioid subtype [19] and low tumor grade [20-22]. Regarding clinical parameters, only one study reported a significant correlation between Bcl-2 and optimal residual disease [23]. Consisting with the findings of several studies, Bcl-2 is associated with a prolonged survival thus with a good prognosis [20, 22-25]. However, some authors found a significant correlation between Bcl-2 status and primary resistance to chemotherapy [26-28]. In our study, Bcl-2 expression was not associated with any clinicopathological parameter. However, survival was significantly associated with bcl2 status (p = 0.02). This result is in accordance with several reports [20, 22].

A significant inverse correlation between Bcl-2 expression and p53 protein accumulation was found in several types of human cancers, especially malignant ovarian tumors [21]. Baekelandt et al [23] found in a series of 103 patients that Bcl-2 expression by itself was not an independent prognostic factor, but the combination of Bcl-2 and p53 staining was a stronger prognostic indicator than p53 expression alone.

Some trials researching in cancer therapies suggest that gene therapy with wild-type p53 [29, 30] or anti-Bcl-2 [31] could enhance response to chemotherapy. However, translation of these new insights to clinical usefulness remains the ultimate and perhaps the most difficult task of the future.

Data regarding the prognostic significance of ER and PR expression in ovarian carcinoma are limited and clinical value of determining steroid hormone receptors in this malignancy is still controversial [9, 32]. This mainly accounts for different detection methods [9]. Today, immunohistochemistry is considered the method of choice because it allows an exact assignement of ER and PR expression to tissue components of interest [9]. Several studies demonstrated that the expression of PR is an independent indicator of favourable prognosis in ovarian carcinoma [9, 33-35] and significant inverse correlation was demonstrated for patient’s age [9], FIGO stage [9, 34], residual tumor [9] and tumor grading [9, 36]. In addition, PR is correlated with endometrioid histologic type [37]. In this study, we also found that PR expression was significantly correlated to early stage, optimal residual tumor, low grade and the group of non serous carcinomas including mainly endometrioid type. In accordance with our results, most reports found that ER status is not a prognostic factor in ovarian carcinoma and doesn’t correlate with any clinicopathological parameter. Only few studies reported a significant expression of ER in advanced FIGO stage [36]. Munstedt et al [9] demonstrated that the favourable course of PR positive ovarian carcinoma relates primarily to the subgroup ER-/PR+ expressing tumors. This tumor phenotype was associated with better prognosis compared to tumors with other steroid hormone receptors combination profiles. Although there is no single explanation for the effect of steroid hormone receptor expression on prognosis, two hypotheses have been proposed: 1) Estrogen-responsive cells efficiently repair DNA and avoid apoptosis, leading to clonal expansion and drug resistance [38]; 2) Progesterone promotes cell differentiation and apoptosis and stimulation of PR inhibits DNA synthesis and cell division [39].

Our results showed a significant correlation between ER and PR: they were both detected or both absent. We didn’t study the ER/PR receptor combination because of the limited number of patients. Apart from prognosis, the predictive role of ER and PR expression with respect to hormonal therapy has not been confirmed [40]. In our study, survival was significantly worse with ascites, positive peritoneal cytology, late FIGO stage, residual lesions after cytoreductive surgery and negative bcl-2 status.

In conclusion, P53, Bcl-2 and hormone receptors status are potential prognostic factors in ovarian cancer. In this study about 57 Tunisian patients with ovarian carcinoma, biomarkers expression was evaluated by immunohistochemistry and was observed in 42 cases (73.7%) for p53, 27 cases (47.4%) for Bcl-2, 20 cases (35.1%) for ER and 19 cases (33.3 %) for PR. Our results showed that p53 expression correlates with aggressiveness parameters such as advanced stage, ascites and positive cytology; whereas PR is associated with favourable prognostic parameters such as early stage, non serous histologic type and low tumor grade. Bcl2 status was significantly associated with better survival.

## Abbreviations

ER: Estrogen receptor
PR: Progesteron receptor
WHO: World Health Organisation
FIGO: International Federation of Gynaecologists and Obstetrics
